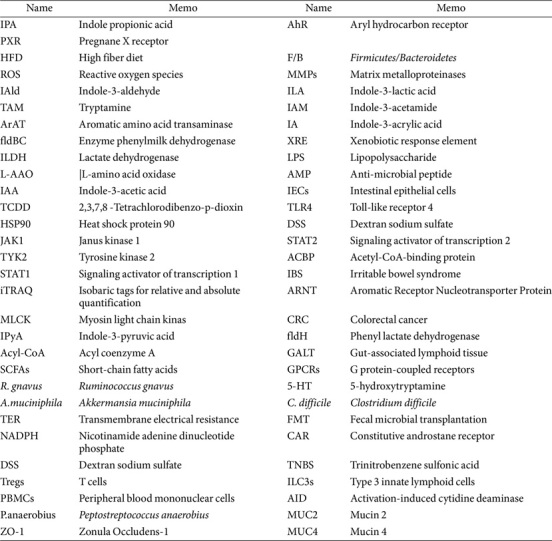# Indole Propionic Acid Regulates Gut Immunity: Mechanisms of Metabolite-Driven Immunomodulation and Barrier Integrity

**DOI:** 10.4014/jmb.2503.03045

**Published:** 2025-08-18

**Authors:** Tao Ren, Dihao Li, Feng Sun, Lijia Pan, Ao Wang, Xinze Li, Yuwen Bao, Meiyu Zhang, Fei Zheng, Hao Yue

**Affiliations:** Jilin Ginseng Academy, Changchun University of Chinese Medicine, Changchun 130117, P.R. China

**Keywords:** Indole propanoic acid, intestinal immunity, microbiome-derived metabolites, AhR/PXR, gut barrier integrity

## Abstract

Indole propionic acid (IPA) is a functional indole derivative produced exclusively by intestinal flora through tryptophan metabolism. Numerous studies have shown that IPA has a variety of beneficial biological functions, including anti-inflammatory and antioxidant effects, immunomodulation, intestinal barrier protection, regulation of intestinal flora composition, and neuroprotection. IPA, as an intestinal microbial metabolite, actively participates in the establishment of intestinal immune homeostasis and positively influences the prevention and control of intestinal diseases, thereby playing an indispensable role in regulating host health. We conducted a comprehensive literature review to explore the synthesis of IPA *in vivo*, the mechanism of action on intestinal immunity, and the promise of its application in the treatment of related diseases. The physiological and biological effects of IPA were investigated to explore its potential application in future drug discovery. Obviously, IPA plays an important role in intestinal immunity and is effective in the treatment of related diseases. IPA helps regulate intestinal immune cell function, inhibiting inflammatory response and enhancing intestinal barrier function through its effects on the aryl hydrocarbon receptor, the pregnane X receptor, and other related signaling pathways. The development of IPA as a target drug for the treatment of intestinal diseases is promising. Although IPA research is still in the experimental animal model stage, there is growing interest in the many therapeutic applications of IPA and increasing opportunities to further modify IPA for future clinical applications.

## Introduction

The intestines are not only the body’s primary site for digestion, nutrient absorption, and metabolism but also its largest immune organ [1, 2]. Indeed, approximately 70–80% of immune cells reside within the gut-associated lymphoid tissue (GALT, hereafter, abbreviations provided in Table 1), highlighting the gut’s critical role in systemic immunity [3]. This specialized immune function emerges from dynamic host-microbiome interactions, where commensal bacteria and other gut microorganisms collectively establish a resilient, interdependent ecosystem that co-regulates host immunity through continuous crosstalk [4, 5]. The intestinal flora and the immune system share a symbiotic (independent and interactive) relationship with intestinal immunity, maintaining homeostasis through the co-regulation of gut microbiota and the host [6]. Host-microbiota immune system crosstalk is orchestrated through multiple molecular mediators, with gut microbial metabolites serving as pivotal immunomodulators that bridge microbial ecology and host physiology [7, 8]. These metabolites are crucial for controlling gut immunological homeostasis, repairing intestinal health, and influencing host physiological and pathological processes [9]. The gut microbiota generates diverse bioactive metabolites with distinct immuno-modulatory functions [10], among them, short-chain fatty acids (SCFAs) such as propionic acid [11] and butyric acid [12], which can inhibit inflammatory responses while contributing to the maintenance of intestinal homeostasis. Bile acids also regulate metabolism by binding to G protein-coupled receptors (GPCRs) and/or nuclear receptors [13-15]. Tryptophan metabolism generates multiple bioactive compounds through both host and microbial pathways, including kynurenine, 5-hydroxytryptamine (5-HT), and various indole derivatives [16, 17]. These metabolites collectively enhance intestinal mucosal barrier integrity and promote overall host health through distinct mechanisms [18].

Tryptophan and its metabolic derivatives serve as crucial signaling mediators for host-gut microbial crosstalk [19, 20]. Depending on how they are catabolized and metabolized in the body, tryptophan metabolites can be categorized as endogenous or microbial-derived [21]. Microbiota-derived tryptophan metabolites are mainly indoles and their derivatives. In the presence of gut microbiota, indole and its derivatives, such as indole, indole-3-aldehyde (IAld), indole propionic acid (IPA), indole-3-lactic acid (ILA), indole-3-ethanol, tryptamine (TAM), indole-3-acetamide (IAM), and indole-3-acrylic acid (IA) contribute to gastrointestinal function and immune system regulation [22-24]. Among them, IPA has attracted much attention due to its unique biological activity. IPA biosynthesis is strictly dependent on intestinal microbiota composition and metabolic activity, with its physiological concentration being precisely regulated through the interplay of dietary intake, microbial community structure, and host metabolic processes [25-28]. However, the specific mechanism of IPA synthesis and its interaction with the intestinal flora have not been fully elucidated. In the following, we have mainly reviewed the effects of IPA, a microbial tryptophan metabolite, on host intestinal immunity and explored its potential future clinical applications. The goal is to provide insights and references for IPA-mediated immune regulation, animal disease treatment, and overall health protection.

## IPA: Intestinal Microbial Metabolite of TRP

### IPA Formation in the Intestine

**Fig. 1:**
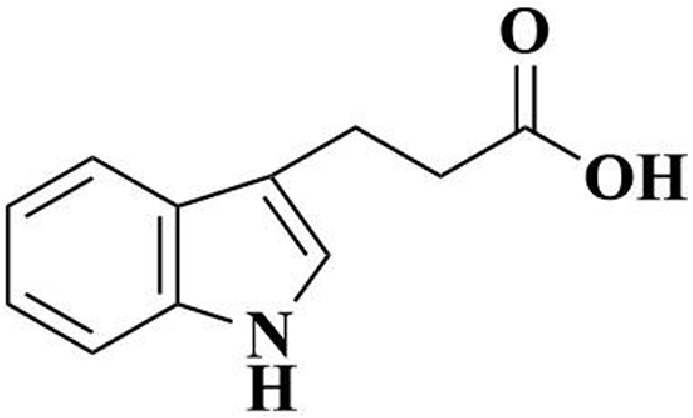
Structural formula of indole propionic acid (IPA).

IPA, also known as 3-indolepropionic acid, indole-3-propionic acid, and azapropionic acid, was first discovered in 1923 (Fig. 1) [29-31]. IPA is one of the tryptophan derivatives metabolized by intestinal gram-positive bacteria (*e.g.*, *Lactobacillus* and *Clostridium perfringens*). It exhibits various physiological effects, such as immune cell function regulation, free-radical scavenging, intestinal barrier function enhancement, intestinal flora regulation, and neuroprotection [30].

**Fig. 2:**
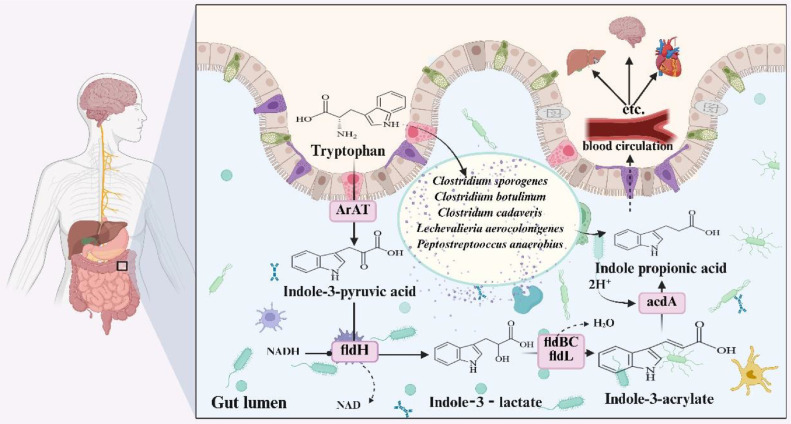
Image illustrating the biosynthesis of indole propionic acid (IPA) *in vivo*. Tryptophan is used to produce IPA in the intestines by enzymes such as aromatic amino acid transaminase (ArAT), phenyllactate dehydrogenase (fldH), enzyme phenylmalate dehydrogenase (fldBC), Acyl coenzyme A (Acyl-CoA), and bacteria such as *Prevotella russellii*, *Peptostreptococcus anaerobius*, and *Porphyromonas stomatis*. IPA acts on multiple organs throughout the body via the blood circulation.

In humans and animals, IPA is synthesized exclusively from tryptophan, which is metabolized by intestinal bacteria [32]. IPA modulates intestinal immunity through multiple mechanisms [33]. The biosynthesis of IPA begins when tryptophan is catalyzed by aromatic amino acid transaminase (ArAT) into indole-3-pyruvic acid (IPyA). IPyA is then converted into ILA by phenyl lactate dehydrogenase (fldH), with IPyA serving as the precursor of ILA. ILA can be produced by dehydrating *Prevotella russellii*, *Peptostreptococcus anaerobius* and *Porphyromonas stomatis* to IA in a process involving the enzyme phenylmalate dehydrogenase (fldBC) and its activator fldH. Acyl coenzyme A (Acyl-CoA) dehydrogenase then converts IA to IPA, the end product of the reductive metabolism of tryptophan (Fig. 2) [34-36].

### Role of Gut Microbiota in the Formation of IPA

Studies in both humans and animals have shown that IPA has a strong interaction with the intestinal flora. Representative IPA-producing strains include *Clostridium sporogenes*, *Anaerostipes hadrus*, and *Anaerobutyricum hallii* [37]. These bacteria carry the complete tryptophan metabolic pathway genes (K00128/K23876/K00179) that synthesize IPA efficiently [35]. *Streptococcus agalactiae* and *Peptostreptococcus anaerobius* (*P. anaerobius*) are capable of independent tryptophan metabolism and IPA production [35, 38, 39]. For instance, *Lactobacillus lactis* can metabolize tryptophan via ArAT and indole lactate dehydrogenase (ILDH) to produce IPyA and ILA [40-42]. Some *Bifidobacterium* spp. can also metabolize tryptophan to produce ILA [43, 44]. The aerobic colonic bacterium *Lechevalieria aerocolonigenes* uses the enzyme L-amino acid oxidase (L-AAO) to break down tryptophan and create IPyA [45]. Recent evidence suggests that *Intestinimonas* and *Ruminococcus gnavus* (*R. gnavus*) may bypass the classical step and utilize unique enzyme systems to directly convert tryptophan or its derivatives to produce IPA [23, 35, 46-49]. Although *Akkermansia muciniphila* (*A. muciniphila*) does not directly synthesize IPA, it indirectly enhances tryptophan metabolism in IPA-producing *Clostridium* species through acetic acid consumption [50]. By modulating the gut metabolic environment, *A. muciniphila* creates favorable ecological conditions that promote the capacity of *Clostridium* to convert tryptophan into IPA [51, 52]. Thus, the production of IPA in organisms has been demonstrated to be co-regulated by various bacterial species.

Studies have shown that shifts in the composition of the gut microbiota can regulate the homeostatic levels of IPA within the host. IPA is primarily synthesized by *Clostridium difficile* (*C. difficile*) in the gut via the tryptophan metabolic pathway [53]. Broad-spectrum antibiotic treatment decreases the relative abundance of *C. difficile* and almost completely inhibits IPA synthesis, reducing levels to nearly zero. In the course of fecal microbial transplantation (FMT) or co-habitation with untreated mice, the intestinal flora underwent a gradual recovery, accompanied by the restoration of IPA levels [50, 54]. However, antibiotic-induced microbiota depletion followed by IPA supplementation only partially rescued anti-infection capacity, suggesting that synergistic interactions with other microbial metabolites are essential for achieving full immunomodulatory effects. The enzyme system composed of K00128, K23876, and K00179 is mainly distributed in anaerobic bacteria of the order *Clostridium* (*e.g.*, *Clostridium sporogenes*, *Anaerobutyricum hallii*), and its community abundance directly determines the efficiency of IPA synthesis [35]. If either enzyme is missing from the microbial community, it will result in disruption of IPA synthesis. When only K00128 and K23876 are present, there is accumulation of indole-3-acetic acid (IAA) with possible conversion to indole and activation of AhR, but no mitochondrial protective effect of IPA [55]. If only K00179 is present, there is an inability to initiate tryptophan metabolism and zero IPA synthesis [56]. It has been observed that certain *R. gnavus* strains are deficient in this system yet nevertheless produce IPA through the substitution of reductases, such as nicotinamide adenine dinucleotide phosphate (NADPH)-dependent enzymes [57]. This observation underscores the flexibility of the pathway.

IPA was positively correlated with some *Lactobacillaceae* and *Bifidobacteriaceae* species (*Limosilactobacillus reuteri*, *Lactobacillus animalis*) and negatively correlated with *Bacteroidales* bacterium M7, viral load, and inflammation markers [38, 58]. The supplementation of IPA in diseased animals has been demonstrated to reduce viral load, decrease local and systemic inflammation, increase the abundance of beneficial bacteria, and improve intestinal microecology [59]. IPA can reduce inflammation by decreasing hypoxia-ischemia-induced elevation of reactive oxygen species (ROS) and activation of matrix metalloproteinases (MMPs) in rat brain microvascular endothelial cells [60]. Treatment of mice with antibiotics targeting IPA-producing bacteria before infection enhanced viral load and lung inflammation, an effect inhibited by IPA supplementation [59]. However, there is a paucity of human trials in this area.

According to medical clinical research, the variety of gut microbes and blood IPA concentration are highly and favorably connected [61]. Specifically, increasing the concentration of circulating IPA in the blood can improve the intestinal flora structure. Both the growth of dangerous bacteria and the multiplication of beneficial bacteria are inhibited by IPA. In rats, the intestinal flora abnormalities brought on by a high-fiber diet (HFD) were considerably alleviated by IPA supplementation administered by gastric gavage, which reversed the increase of *Firmicutes*/*Bacteroidetes* (F/B), and increased the abundance of *Oscillibacter* spp. and *Odoribacter* spp. [62-64]. Yin [65] added IPA (5-50 μmol/l) to *A. muciniphila* BAA-835T and then anaerobically incubated it at 37°C for 24 h, which significantly promoted the growth of the bacterium. The best effect was achieved when the IPA concentration was 25 μmol/l. The study noted that IPA might promote the growth of *Akkermansia* by influencing its division. As a novel probiotic, *Akkermansia* has drawn a lot of interest because it can help treat and lessen a number of metabolic disorders, including diabetes and obesity. Negatu [66] showed that intragastric administration of IPA (100 mg/kg, BW/d) could significantly reduce the abundance of *Mycobacterium tuberculosis* caused by a respiratory infection in mice, indicating that IPA has anti-*M. tuberculosis* activity. Furthermore, *in vitro* tests have shown that IPA can stop the growth of a number of dangerous bacteria, including *Salmonella* Typhimurium and *Legionella pneumophila*, respectively [67, 68].

The majority of contemporary studies utilize sterile or antibiotic-treated mice, which may not fully replicate the complexity of human microbial communities. Host genetic polymorphisms (*e.g.*, pregnane X receptor (PXR)/constitutive androstane receptor (CAR) variants) may modulate the efficacy of IPA; however, this aspect has not been thoroughly investigated [69]. Targeted modulation of these flora or enzyme activities may be a novel strategy to intervene in intestinal immunity. However, the exact mechanisms by which IPA inhibits and scavenges harmful bacteria *in vitro* and *in vivo* remain unclear. One hypothesis suggests that IPA functions similarly to indole and may inhibit the growth of harmful bacteria by interfering with the population-sensing signals of bacteria or inhibiting the production of virulence factors. Therefore, further studies are needed to elucidate the exact mechanism of how IPA regulates and improves the gut microbiota.

## IPA Regulates Intestinal Immunity *via* AhR/PXR Pathways

IPA has been established as a dual-function microbial metabolite capable of modulating both aryl hydrocarbon receptor (AhR) and PXR signaling pathways [70, 71]. This substance has been demonstrated to maintain intestinal immune homeostasis and prevent intestinal inflammation by regulating the intestinal barrier, influencing immune cell function and differentiation, and regulating inflammatory factors [72-75]. A comprehensive understanding of the role and mechanism of IPA in regulating intestinal cellular immunity and inflammatory response is imperative for enhancing intestinal health.

## Regulation of the Intestinal Barrier

### The AhR Pathway

IPA has been identified as a medium-affinity ligand (EC50 ≈ 10-50 μM) for the AhR pathway [42]. Upon binding to IPA, heat shock protein 90 (HSP90) dissociates from AhR, facilitating its translocation from the cytoplasm into the nucleus [76]. There, it forms a heterodimer with the aryl hydrocarbon receptor nuclear translocator (ARNT), which binds to xenobiotic response elements (XREs) and modulates the expression of downstream genes [77]. For instance, the anti-inflammatory genes *IL-10* and *CYP1A1*, metabolic enzymes like CYP1A1, as well as the genes associated with the barrier function, including Mucin 2 (MUC2) and Occludin, are notable examples [42, 57, 78, 79]. Activation of AhR by IPA enhances the intestinal barrier by upregulating tight junction proteins, including Occludin, Claudin-1, and Zonula Occludens-1 (ZO-1), thereby reinforcing epithelial integrity, reducing intestinal permeability, and inhibiting lipopolysaccharide (LPS) translocation [80]. Additionally, IPA promotes mucin secretion (*e.g.*, MUC2) from goblet cells, contributing to the structural and functional reinforcement of the mucus layer [81, 82]. Li [80] demonstrated these effects using a Caco-2/HT29 co-culture model. IPA significantly upregulated the mRNA and protein expression of Claudin-1, Occludin, and ZO-1, even under LPS-induced inflammatory conditions. Moreover, IPA markedly increased the expression of MUC2 and Mucin 4 (MUC4), confirmed by both western blot and immunofluorescence staining.

In intestinal epithelial cells, this pathway has been shown to significantly enhance the expression of barrier function-related genes, including Occludin and Claudin-4 [78]. The experimental data [62] demonstrated that IPA treatment (50 μM) led to a significant upregulation in the expression of tight junction proteins in human colonic organoids: The expression levels of Occludin, Claudin-1, and ZO-1 were found to be significantly elevated. Occludin exhibited an increase of 3.2-fold, Claudin-1 increased by 2.8-fold, and the expression of ZO-1 increased by 4.1-fold. The transmembrane electrical resistance (TER) of Caco-2 monolayers exhibited a 65%increase, indicative of a substantial enhancement in intestinal barrier function. HDAC3 removes transcriptional activation marks by deacetylating H3K27ac/H4K16ac histones, which results in localized chromatin tightening [83]. This “repressive” modification enhances the opening of promoter regions of AhR target genes (*e.g.*, *CYP1A1* and *IL-22*). The “pro-transcriptional” effect of *HDAC3* is gene-specific and may apply only to AhR target genes.

### The PXR Pathway

**Fig. 3:**
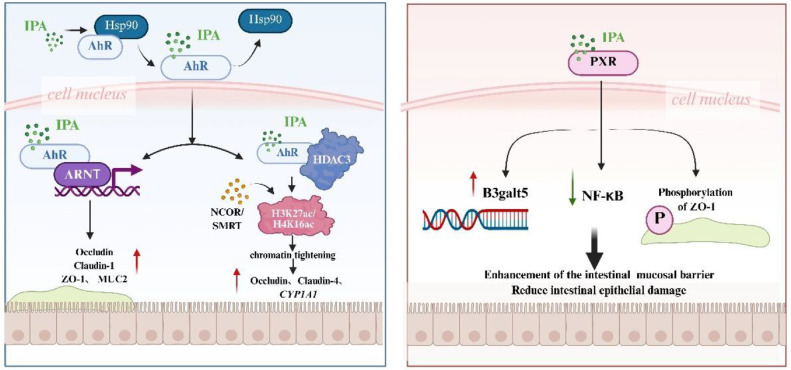
Indole propionic acid (IPA) regulates the intestinal barrier through Aryl hydrocarbon receptor (AhR)/Pregnane X receptor (PXR). IPA upregulates the expression of proteins such as Occludin, Claudin-1, Zonula Occludens-1, Mucin 2, and Mucin 4 through activation of AhR. IPA also strengthens the expression of the B3galt5 gene through activation of PXR, inhibits NF-κB signaling, and enhances mucin expression, which in turn maintains the intestinal barrier.

IPA also serves as a ligand for the PXR, a nuclear receptor highly expressed in intestinal tissues [54, 84]. PXR plays a pivotal role in maintaining intestinal homeostasis through its regulation of microbial metabolism, immune responses, and barrier integrity [85]. IPA triggers the following protective mechanisms by directly binding to the PXR ligand-binding domain (Kd = 8.7 μM) [86]. IPA-induced PXR activation has been shown to enhance mucin glycosylation by upregulating B3galt5, a gene involved in O-glycosylation processes, thereby strengthening the mucosal barrier [87]. Organoid experiments showed that IPA treatment increased the thickness of the mucus layer from 15 ± 2 μm to 28 ± 3 μm [80]. Additionally, PXR modulates inflammatory responses via the TLR4/NF-κB signaling axis [88]. Evidence from murine models of dextran sodium sulfate (DSS)- and trinitrobenzene sulfonic acid (TNBS)-induced colitis demonstrates that oral IPA supplementation ameliorates epithelial injury and inflammation in a PXR-dependent manner, and IPA-PXR enhances tight junction integrity by ZO-1 phosphorylation [62]. Studies also show that IPA-treated mice exhibit improved epithelial integrity, increased villus height, and decreased systemic inflammation through the PXR/ACBP signaling pathway [89]. In contrast, *Nr1i2* knockout (Nr1i2^-^/^-^) mice display severe mucosal barrier damage, supporting the essential role of IPA–PXR signaling in barrier protection (Fig. 3) [87].

## Regulation of Immune Cells

### The AhR Pathway

Due to its widespread expression in intestinal immune cells, AhR is a central target for IPA to modulate mucosal immunity. IPA, a microbial tryptophan metabolite, orchestrates pro- and anti-inflammatory responses in a cell-type-specific manner by binding to the AhR expressed by various immune cell populations, including Th17 cells, regulatory T cells (Tregs), type 3 innate lymphoid cells (ILC3s), macrophages, and B cells [9]. Mechanistic studies have demonstrated that an IPA concentration of 20-50 μM promotes Treg differentiation through AhR-mediated binding of RORγt regulatory elements, while inhibiting Th17 polarization through HDAC3-dependent inhibition of the STAT3 signaling pathway [90]. This results in a significant decrease in the proportion of Th17 cells in lamina propria lymphocytes, from 18.5% to 12.8% (*p* < 0.001) [91]. Notably, the AhR antagonist CH223191 significantly reduced the increase in Treg differentiation induced by IPA in peripheral blood mononuclear cells (PBMCs), suggesting a direct, AhR-dependent mechanism [92]. Meanwhile, IPA enhanced IL-22 production by ILC3s through circadian-regulated AhR activation, which increased by 3.5-fold. This increase was particularly pronounced in the small intestinal subpopulation, where single-cell RNA sequencing demonstrated a 4.1-fold increase in AhR-targeted gene induction compared to colonic ILC3s [30]. IPA’s immunomodulatory capacity extends to innate immunity. It inhibits NLRP3 inflammasome activation in macrophages (55% reduction in caspase-1 activity) and promotes IgA class switching in B cells by upregulating activation-induced cytidine deaminase (AID) (3.2-fold increase) [93]. Clinical observations in patients with ulcerative colitis revealed impaired IPA-AhR signaling, evidenced by a 42% reduction in mucosal AhR expression and decreased fecal IPA levels (2.1 μM vs. 5.8 μM in healthy controls)[94]. These levels were negatively correlated with disease severity. While these results suggest the therapeutic potential of targeting the IPA-AhR axis, significant challenges, such as poor colonic bioavailability (less than 15%absorption) and variations in individual microbial IPA production, currently hinder its clinical application.

### The PXR Pathway

**Fig. 4:**
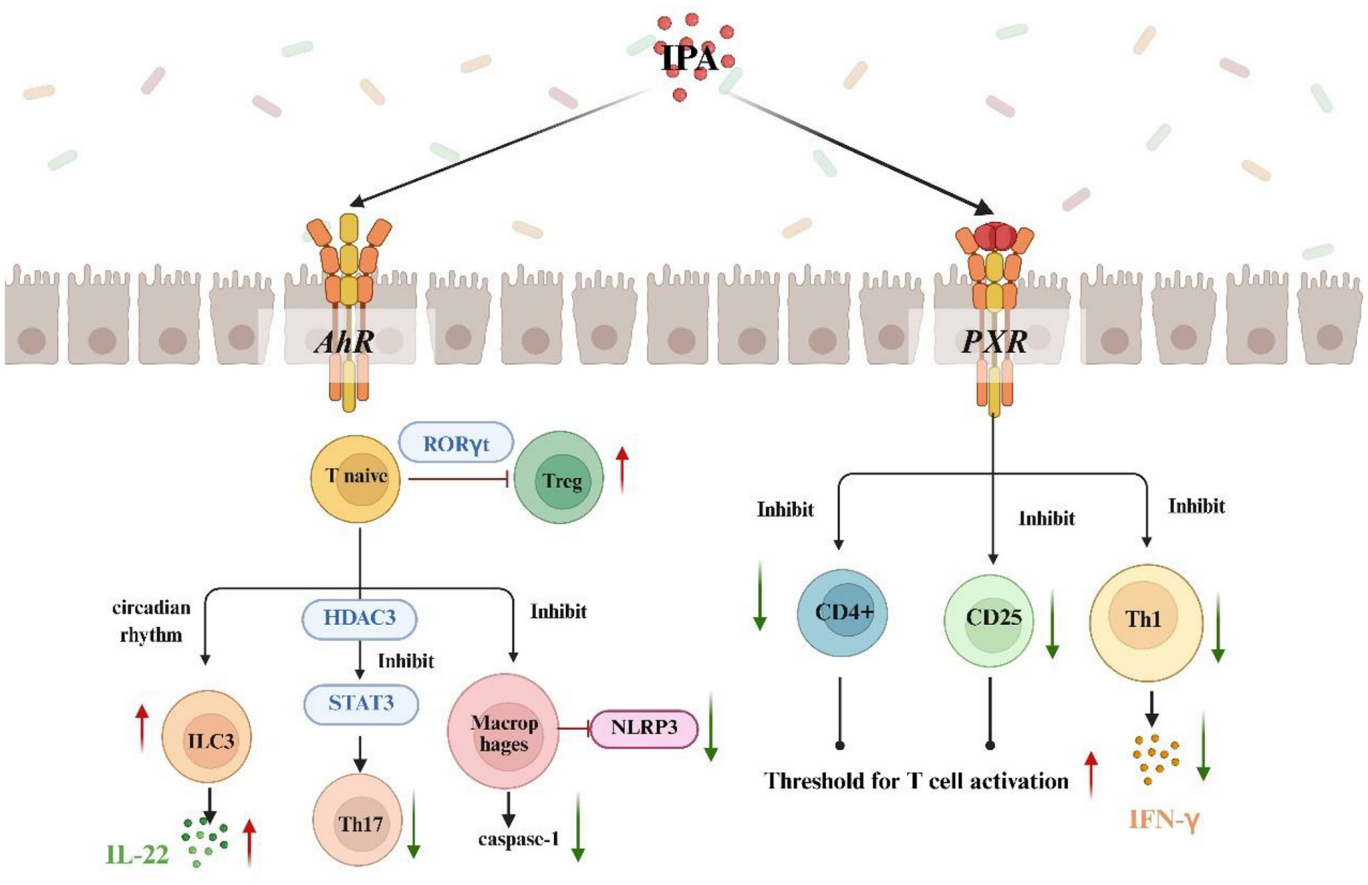
Indole propionic acid (IPA) regulates intestinal immune cells through Aryl hydrocarbon receptor (AhR)/Pregnane X receptor (PXR). IPA can regulate the status of Tregs, innate lymphoid cells type 3 (ILC3), Th17 cells, and NOD-like receptor family pyrin domain-containing 3 (NLRP3) via AhR. IPA can inhibit the expression of CD4+/CD25/ Th1 via PXR.

As a nuclear receptor predominantly expressed in intestinal epithelial cells and immune cells, PXR serves as a key mediator of IPA’s immunosuppressive actions, particularly in modulating T cell responses [95]. Mechanistic studies demonstrate that IPA (50-100 μM) significantly suppresses CD4+ T cell proliferation (40-60% reduction) and downregulates activation markers including CD25 and IFN-γ through PXR-dependent pathways, as evidenced by complete reversal of these effects in PXR knockout models [91, 96, 97]. In intestinal epithelial cells, IPA-PXR signaling plays a crucial role in maintaining barrier function by reversing IFN-γ-induced expression of the fructose transporter GLUT5 (70% reduction) and preventing inflammation-associated increases in paracellular permeability (TER restoration to 80% of baseline) [98]. The role of PXR in regulating immunosuppression is further demonstrated by the fact that PXR deletion is associated with an enhanced Th1-type immune response, which is normalized by IPA treatment [96, 99]. Nevertheless, the extent to which IPA directly influences immune cell subsets via PXR remains an open question requiring further investigation (Fig. 4).

## Regulation of Cytokines

### The AhR Pathway

IPA–AhR signaling modulates cytokine profiles, enhancing the expression of IL-22 and IL-10 while suppressing IL-17A [100, 101]. The AhR–IL-22 axis plays a vital role in maintaining mucosal immunity, promoting antimicrobial peptide secretion, and regulating microbiota composition [102, 103]. Hou [104] reported that IAld, another microbial metabolite, activated AhR in innate lymphocytes, induced STAT3 phosphorylation, and stimulated IL-22 secretion, thereby facilitating mucosal healing. AhR activation by IPA also attenuates Th17 differentiation via inhibition of the STAT3 and RORγt pathways [90]. Sonia Mohanta [105] found that AhR-/- mice exhibited a pronounced Th17 response in a DSS-induced colitis model. This response was ameliorated by IPA supplementation, resulting in lower IL-17A and IL-22 levels and reduced colonic inflammation. Moreover, IPA enhances macrophage phagocytic activity through AhR-mediated modulation of STAT3 and NF-κB signaling [106]. The protective effect of IPA was significantly diminished by AhR inhibition, highlighting its essential role in cytokine regulation and immune protection.

### The PXR Pathway

**Fig. 5:**
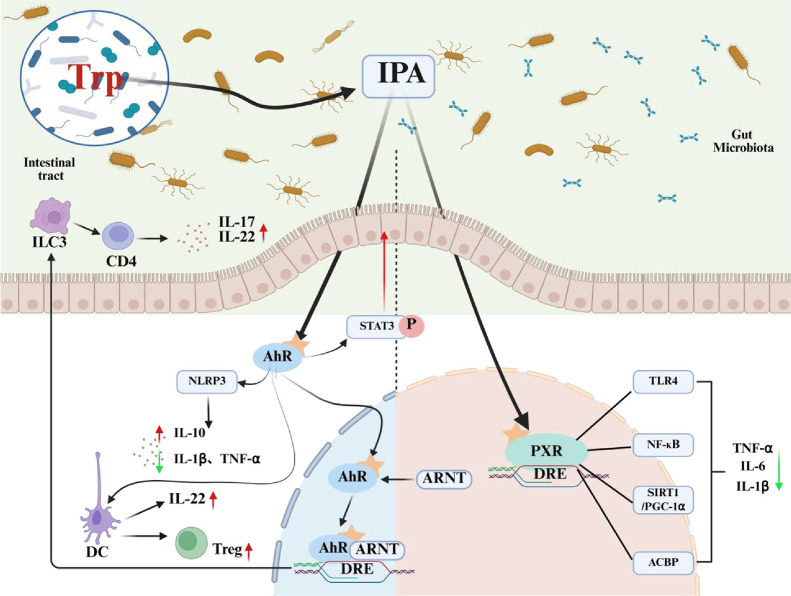
Indole propionic acid (IPA) affects cytokines through Aryl hydrocarbon receptor (AhR)/Pregnane X receptor (PXR). IPA regulates immune responses and metabolic pathways, as well as influencing cytokine secretion, by activating the AhR and PXR in intestinal epithelial cells.

IPA-induced PXR activation also exerts profound effects on cytokine expression. PXR inhibits the transcription of TNF-α, IL-6, and IL-1β by suppressing canonical NF-κB signaling [107, 108]. This immunoregulatory mechanism has been validated in both epithelial cells and macrophages. Additionally, PXR modulates the expression of metabolic-immune crosstalk genes, such as *CYP3A4*, *ABCB1*, and *NR0B2*, which may contribute to systemic anti-inflammatory effects and metabolic regulation (Fig. 5) [109].

## IPA Improves Intestinal Health

Intestinal immunity is closely linked to intestinal health. Once disrupted, it can trigger chronic microinflammation, leading to severe intestinal damage [110]. Recent studies, bolstered by a significantly improved understanding of IPA’s biological role, highlight IPA as a promising therapeutic approach for intestinal diseases. The main intestinal diseases are colitis, inflammatory bowel disease (IBD), colorectal cancer (CRC), and irritable bowel syndrome (IBS). IBS has been found to be significantly correlated with dysbiosis of the intestinal flora [111]. Conversely, the evolution of colitis into IBD and subsequent progression to CRC is a multistep chronic inflammatory-cancer process. These findings position IPA as a multifaceted regulator of barrier homeostasis, bridging inflammation control and tissue repair—a recurring theme in other intestinal disorders.

### IPA Improves Colitis

The incidence of colitis is rising in China and is characterized by chronic, recurrent, and disabling inflammation with no definitive cure [112-114]. Although the pathogenesis is unclear, damage to the intestinal barrier and repair dysfunction are basic pathological changes and disease characteristics of colitis [115, 116]. Hanju Lee used the amino acid conjugation of IPA to prepare a colon-targeted indole propionic acid prodrug, which can effectively treat colitis in rats and enhance its anti-colitis activity through the synergistic delivery of the *Hcar2* agonist 5-ANA [117]. Additionally, IPA resists radiation-related toxicity by binding PXR to increase the expression of ACBP [118]. Xiao [89] used isobaric tags for relative and absolute quantification (iTRAQ) analysis and found that IPA reprogrammed radiation-induced changes in the protein expression profile of small intestinal tissue, including ACBP. *In vitro* and *in vivo* experiments, such as intracellular PXR/ACBP interference with intracellular PXR/ACBP knockout studies in mouse intestinal cells using hydrodynamic transfection plasmid sh-ACBP/PXR, show that IPA cannot mitigate the suppression of cell proliferation caused by ionizing radiation when ACBP is disrupted. Additionally, the loss of ACBP in small intestinal tissues of mice exacerbates intestinal damage, leading to a shortened colon, increased inflammation, elevated ROS levels, decreased number of intestinal villi and cupped cells, and deterioration of gastrointestinal tract function and epithelial integrity [89].

The above studies provide new therapeutic ideas for targeted delivery of IPA to enhance anti-inflammation in colitis. However, these findings are still at an early stage, and more clinical trials and in-depth studies are needed to validate the efficacy and safety of IPA. Future studies should focus on unraveling the specific mechanism of action of IPA, evaluating its efficacy and safety in different populations, and exploring its potential application in other intestinal diseases.

### Indoles Relieve IBD

The persistent antigenic stimulation of colitis is the root cause of an irreversible imbalance in immune homeostasis. This imbalance ultimately leads to a pathologic autoimmune response against intestinal commensal bacteria, resulting in the chronic relapsing inflammation that is characteristic of IBD. IBD is a chronic, recurrent, and nonspecific inflammatory disease of the intestinal tract with an unclear etiology. Its pathogenesis is related to genetic susceptibility, environmental factors, intestinal microecology and immune disorders [119, 120]. Numerous studies have shown that metabolic derivatives of tryptophan increase levels of tight junction proteins to enhance intestinal barrier function [57, 121, 122]. IBD is linked to the mucus layer, and IBD patients have a thinner internal mucus layer and lower MUC2 glycosylation [123]. Serum and fecal samples from IBD patients have been reported to have severely reduced IPA concentrations compared to serum and fecal samples from healthy individuals [124]. In addition, studies in mice lacking the IBD susceptibility gene have demonstrated significantly increased AhR activation and MUC2 gene expression. Even in LPS-stimulated co-cultures, IA maintained its effect on MUC2 gene expression and increased IL-10 production [38]. MUC production by thrush cells depends on IL-10; therefore, bacterial production of IA has the potential to increase IL-10 production and *MUC* gene expression, which may be beneficial to patients with IBD [125]. Thus, IPA is biologically important in the tryptophan metabolic pathway as the final metabolite of IA.

LPS decreased the expression levels of TEER and tight junction proteins in NCM460 cells; however, IPA treatment attenuated the effects of LPS, highlighting the protective effect of IPA against LPS-induced intestinal barrier dysfunction *in vitro* [81]. In addition, studies in mice lacking the IBD susceptibility gene *Card9* have shown that a lack of microbiota capable of producing AhR agonists is associated with an increased risk of colitis [126]. These data suggest that delivery of adequate AhR agonists to the gut may be a promising technique for the treatment of IBD [127]. Indole analogs such as IPA have proven to be potent AhR activators and may be effective immunomodulatory therapies for the treatment of IBD patients [106]. After IPA administration, an increase in the proportion of M2 macrophages is observed [128]. In IBD, immune cells tend to express low AhR levels and activity due to reduced concentrations of AhR ligands derived from the gut microbiota [129]. However, how IPA affects the composition and function of the gut microbiota and how these changes in turn affect the host immune response has not been elucidated, and more in-depth studies are needed with a view to early clinical application.

### IPA Remits CRC

The persistent inflammatory microenvironment in IBD has been shown to cause epithelial cells to evade immune surveillance and accumulate oncogenic mutations by inducing genomic instability and immunoediting. Ultimately, this process leads to the irreversible transformation of the intestinal mucosa from chronic inflammation to malignancy, which is the core pathogenesis of IBD-associated CRC. An increasing body of data suggests that intestinal tryptophan metabolites, especially indoles, apparently play an important role in CRC [130]. Inhibition of the indole-AhR signaling pathway has been shown to significantly increase mRNA levels of TNF-α, IL-1β, and IL-6 in a model of inflammation-related colorectal carcinogenesis [131]. Additionally, indole has been found to stimulate the synthesis of the anti-inflammatory cytokines IL-10 and IL-22. Moreover, indoleamine 2,3-dioxygenase (IDO1), an enzyme involved in tumor immunological tolerance, is inhibited by the indole derivative tryptamine [131].

Altered microbial tryptophan metabolism is also characteristic of CRC [132]. Compared to healthy individuals, CRC patients have a lower indole/tryptophan ratio and a higher kynurenine/tryptophan ratio [132]. Indole synthesis is reduced while Kyn and IDO1 expression is up in colorectal cancer. Activated T cells experience cell cycle arrest as a result of elevated IDO1 activity and enhanced Trp depletion, which causes apoptotic T cell death, increases immunosuppression, and reduces the generation of indole in the tumor microenvironment [133]. Decreased indole production attenuates the inhibitory effect on colon cancer [132]. In conclusion, altered microbial trp-indole metabolic pathways evidently play a role in CRC pathogenesis. Therefore, determining the role of indoles in colorectal cancer pathogenesis is crucial for the development of possible therapeutic approaches. Thus, while IPA’s barrier-protective effects align with its roles in colitis/IBD, its unique ability to reverse tumor-associated immunosuppression highlights context-dependent actions.

### IPA for IBS

Even in non-inflammatory IBS, IPA demonstrates translational potential. One of the most common gastrointestinal conditions is IBS [134], and epidemiologic studies estimate that IBS affects 9%-14% of adults worldwide [135]. Research suggests that IPA may alleviate IBS symptoms by enhancing the intestinal mucosal barrier and regulating visceral sensitivity. In the IBS-D mouse model, IPA administration has been associated with decreased serum levels of 5-HT, SP, D-LA, DAO, and colonic tissue inflammatory factors IL-6 and TNF-α, ultimately improving the IBS-D symptoms. This suggests IPA's pleiotropy spans from epithelial repair to neural signaling—a unifying thread across intestinal pathologies.

## Discussion

Experimental evidence suggests that IPA, as a gut microbial metabolite, may be a potential target for the treatment of intestinal inflammation and related diseases. However, studies have mainly focused on short-term intervention effects, and lack data on long-term therapeutic efficacy and safety. Moreover, the long-term effects of higher or lower concentrations of IPA on the intestinal barrier have not been evaluated. Meanwhile, the composition and function of the gut microbiota vary significantly between individuals, which may lead to individualized responses to IPA.

The presence of IPA in the cerebrospinal fluid of both humans and rodents suggests similarities in IPA distribution across species. IPA is primarily eliminated through hepatic metabolism and renal excretion. Serum levels of IPA decrease significantly in cases of renal insufficiency, indicating a dependence on renal clearance. Species differences affect IPA metabolism and receptor sensitivity. For instance, mouse PXR is more easily activated by IPA than human PXR. At an IPA concentration of 50 μM, the expression of *Cyp3a11* in mouse intestinal epithelial cells increased eight- to tenfold, while the expression of *CYP3A4* in human-derived cells increased two- to threefold. IPA itself is a relatively weak AhR ligand, and its affinity for human AhR is slightly lower than for mouse AhR. Therefore, the metabolic rate and bioavailability of IPA may differ between humans and rodents [136]. Species specificity must be considered in research and dosing.

The immunomodulatory effects of IPA are clearly dose-dependent. In various animal models, low doses of IPA typically promote immune homeostasis and tolerance. For instance, supplementing a high-salt hypertensive mouse model with 5 mg/kg/day of IPA significantly reduced Th17 cells and increased Treg cells, resulting in improved inflammation and sodium excretion. Conversely, excessive amounts of IPA (50 mg/kg/day) may overactivate AhR, causing immunosuppression. Similarly, high doses of IPA may enhance the IL-10/TGF-β pathway, which generates tolerance and suppresses the immune response. Tables 3 and S1 in the study by Hui Jiang [9] (2022) summarize the dosage and methodology of IPA in animal models and cell lines.

Together, AhR and PXR constitute a dual-pathway regulatory network for immunomodulation by IPA. They are both synergistic and complementary, as well as functionally antagonistic. This complex interplay profoundly affects the final biological effects of IPA. In terms of synergism, AhR and PXR collaborate to maintain intestinal barrier function through spatial complementarity. AhR promotes mucin secretion by cup cells, primarily via the IL-22/STAT3 pathway, which results in a threefold increase in MUC2 expression. PXR, on the other hand, enhances tight junction integrity, increasing transmembrane resistance by 65%, by inhibiting IFN-γ-induced myosin light-chain kinase (MLCK) activation. This synergistic protection was validated in the DSS colitis model, in which AhR/PXR double-knockout mice exhibited more severe epithelial damage than single knockouts (an 80% vs. 40% increase in pathology score, *p* < 0.01). Regarding oxidative stress regulation, the AhR-Nrf2-HO-1 axis establishes a dual defense system alongside PXR-mediated NADPH oxidase inhibition (a 60% decrease in activity). However, PXR gene deletion leads to loss of IPA’s antioxidant capacity.

Furthermore, the two major pathways in immune cell regulation are significantly antagonistic. The AhR promotes Foxp3+ Treg differentiation by directly binding to Foxp3’s conserved non-coding sequences [137]. In contrast, the PXR generally inhibits T cell activation by downregulating CD3-chain phosphorylation. This antagonism results in IPA’s unique dose-dependent effect: AhR dominates at low concentrations (1-10 μM), causing increase in the Treg/Th17 ratio. At high concentrations (>50 μM), PXR takes center stage, inhibiting CD4+ T-cell proliferation and upregulating PD-L1. This “concentration switch” effect was also observed in macrophage polarization. Physiological concentrations of IPA induced M2-type polarization via AhR, while supraphysiological concentrations led to an immunosuppressive phenotype via PXR.

## Conclusion

Current animal experimental data indicate that IPA has significant therapeutic effects in colitis, IBD, and IBS, and can exert its effects through multiple mechanisms, such as improving the structure of the intestinal flora, enhancing the mucus barrier, and reducing the inflammatory response. In particular, in colorectal cancer models, IPA reverses the state of immune tolerance caused by aberrant IDO1 activity by restoring the tryptophan-indole metabolic axis, providing a theoretical basis for cancer immunotherapy targeting metabolic remodeling. Despite the wide range of biological functions of IPA, its research is still at an early stage, mainly focusing on animal models and *in vitro* experiments. The issues of IPA’s dose-dependence, species-specificity, and individualized differences have not been fully resolved. In particular, the potential risks of high doses of IPA, such as immunosuppression, side effects, and over-activation of AhR/PXR signaling, need to be verified by systematic toxicology and preclinical studies. In addition, the specific target cell types of IPA, the dynamic regulatory mechanisms in different immune environments, and the synergistic or antagonistic relationships need further research.

The pathophysiology of diseases is significantly influenced by intestinal bacteria. Therefore, the mechanism of action of IPA needs to be better understood to provide new ideas for disease prevention as well as diagnosis and treatment. IPA, as an active metabolite derived from intestinal flora, is an important factor in regulating host immunity, maintaining intestinal homeostasis, and intervening in a variety of chronic diseases. Future research should strengthen the exploration of the long-term mechanism of IPA action in the human body and promote its translation to the clinic, including the synthesis and optimization of IPA derivatives, the construction of targeted delivery systems, and the combined treatment mode with probiotics or dietary intervention. Along with a deeper understanding of IPA's mechanism of action, its application in the maintenance of intestinal health and the treatment of systemic diseases is highly anticipated.

## Figures and Tables

**Table 1:** Abbreviations.